# Survivorship, complications and patient-reported outcomes in calcar-guided short-stem THA: prospective mid-term multicenter data of the first 879 hips

**DOI:** 10.1007/s00402-022-04354-z

**Published:** 2022-01-25

**Authors:** Karl Philipp Kutzner, Steven Mark Maurer, Ingmar Meinecke, Guido Heers, Dominique Bosson

**Affiliations:** 1 Department of Orthopaedics and Traumatology, St. Josefs Hospital Wiesbaden, Beethovenstr. 20, 65189 Wiesbaden, Germany; 2 Department of Orthopaedics and Traumatology, University Medical Centre of the Johannes Gutenberg-University of Mainz, Langenbeckstr. 1, 55131 Mainz, Germany; 3 Department of Orthopedic Surgery, Bürgerspital Solothurn, Schöngrünstr. 38, 4500 Solothurn, Switzerland; 4 Helios Park-Clinic Leipzig, Strümpellstr. 41, 04289 Leipzig, Germany; 5 Department of Orthopedic Surgery and Arthroplasty, Vitos Orthopaedic Clinic Kassel, Wilhelmshöher Allee 345, 34131 Kassel, Germany; 6 Clinique de Genolier, Route du Muids 3, 1272 Genolier, Switzerland

**Keywords:** Short stem, Total hip arthroplasty, Survival, Complication, Optimys

## Abstract

**Introduction:**

Short stems are a bone and soft-tissue preserving alternative to conventional stems. The aim of this multicenter study is to present the mid-term outcomes of a calcar-guided short stem.

**Materials and methods:**

This is a prospective case series of the first 879 total hip arthroplasties performed on 782 patients across 5 centers using identical calcar-guided short stems. In a mid-term follow-up (6 years), rates and reasons for complications and revisions were documented. The Harris Hip Score (HHS) was obtained; patients reported pain and satisfaction using a visual analog scale.

**Results:**

A total of 43 patients died in the study cohort for non-related reasons; 26 patients (3.0%) required at least 1 revision after the index procedure. The survival rate for endpoint stem revision at mid-term was 98.4%. The main reasons for stem revision were aseptic loosening and early periprosthetic fractures. Sex had no influence on stem survival. Older patients or those with a high body mass index showed increased risk for stem revision during follow-up. Dorr type A morphology revealed a significantly lower risk of stem revision than Dorr type B or C (*p* = 0.0465). The HHS, satisfaction, and load pain at mid-term were 96.5 (SD 8.0), 9.7 (SD 0.9), and 0.5 (SD 1.9), respectively.

**Conclusions:**

This short stem produced highly satisfactory outcomes at mid-term, with 98.4% implant survival for any cause of stem revision and low complication rates. Long-term results are required to further evaluate these promising mid-term results.

## Introduction

Short stems present as a bone and soft-tissue preserving alternative to conventional stems in total hip arthroplasty (THA) and have thus gained popularity in young and active patients over the past decade. However, the short stems implanted are quite heterogeneous, providing distinct differences regarding stem length, level of osteotomy, and insertion technique [1].

Data on the long-term survival rate of short stems are scarce. A recent review of national registry data, together with case studies, found a revision rate of approximately 5% after 10 years for several conservative implants [2]. It is not yet possible to predict whether this new group of stems will perform as well as conventional stems.

**Fig. 1:**
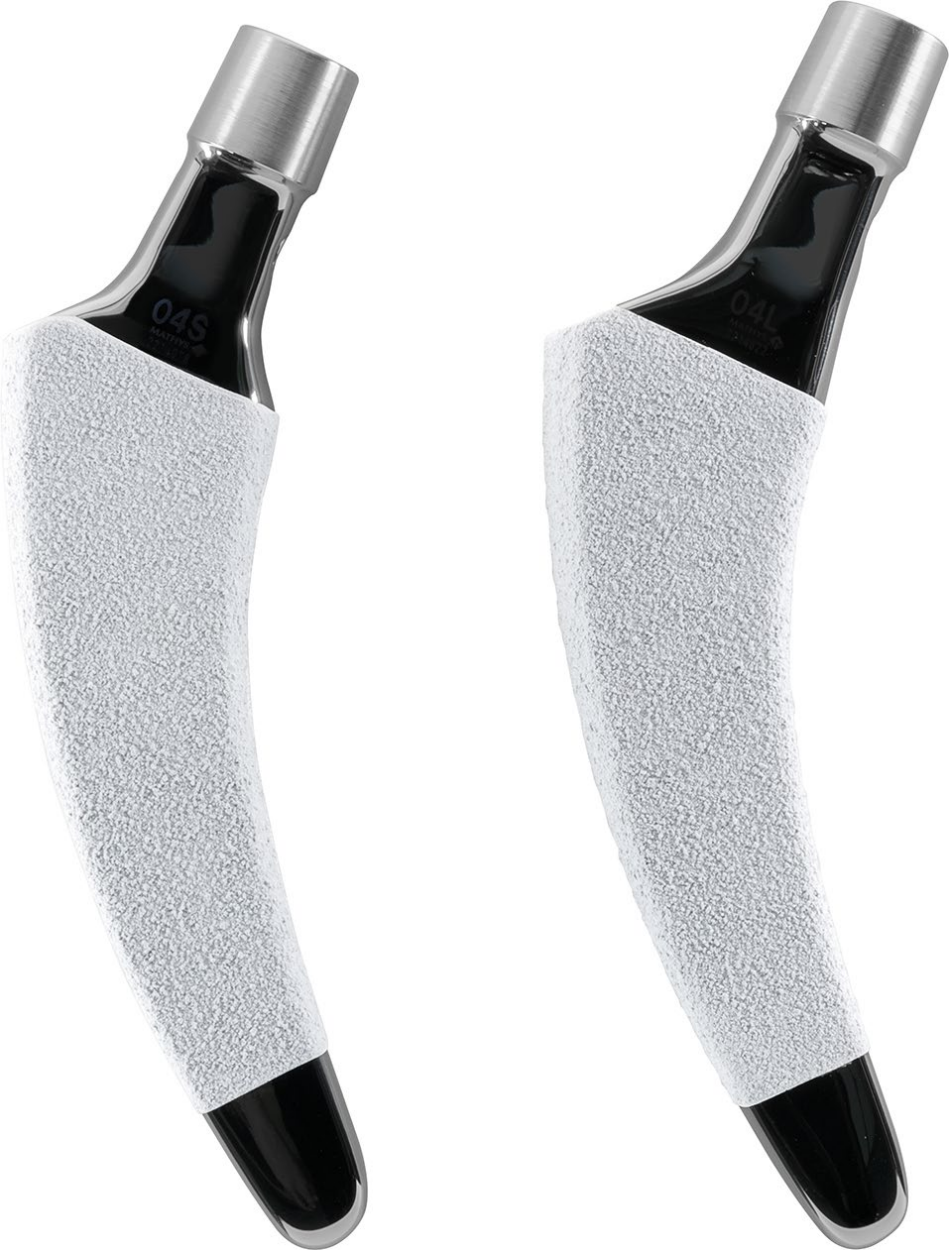
Optimys stem (Mathys Ltd., Bettlach, Switzerland) with two different offset versions (standard and lateral)

The optimys short stem (Mathys Ltd, Bettlach, Switzerland) was introduced in 2010 as part of the current multicenter study and has been available on the international market since 2013 (Fig. 1). This new-generation short stem is designed to allow reconstruction of individual patient anatomy by following the calcar of the femoral neck [3]. It uses meta-diaphyseal anchoring, meaning that fixation uses either pronounced metaphyseal anchorage or additional diaphyseal anchorage, depending on the stem alignment [4, 5]. Thus, classification for this stem design is not easy. Particularly in Europe, the term “calcar-guided” has become established in recent years [6].

A study of the short-term clinical outcome showed excellent patient-reported outcome measures (PROMs); low rates of aseptic loosening or other signs of implant failure were observed at 2 years after implantation [7]. However, mid-term results are scarce for this specific stem design.

The aim of this multicenter study was to assess survivorship and complications of the first 879 implantations of the optimys stem worldwide in a mid-term follow-up analysis. This report updates previously published short-term results [7, 8].

## Materials and methods

The investigators conducted a multicenter observational cohort study according to the STROBE guidelines, including 782 patients, for whom 879 hips were operated on between December 2010 and May 2014 across 5 clinical sites Germany and Switzerland, evaluating clinical results as well as survivorship and complications. The Freiburgethics commission international (FECI)) approved the study (010/2071), written informed consent was obtained of all patients included and it was carried out in compliance with the Helsinki Declaration.

Patients were considered to be eligible for the study if they were adults (age > 18 years), were admitted with a diagnosis of either primary or secondary hip osteoarthritis as well as osteonecrosis of the femoral head and, as a result, were operatively managed with cementless THA using the investigated implant, according to the surgeon’s preference. Femur morphology was assessed for all patients using the Dorr classification (Table 1) [9].

**Table 1:** Distribution of Dorr classification

Clinic	Dorr classification			
	A	B	C	Total
Center 1	149 (69.0%)	66 (30.6%)	1 (0.5%)	216
Center 2	157 (56.5%)	106 (38.1%)	16 (5.4%)	278
Center 3	103 (44.8%)	124 (52.6%)	8 (2.6%)	230
Center 4	29 (34.1%)	52 (61.2%)	4 (4.7%)	85
Center 5	30 (46.9%)	30 (46.9%)	4 (6.3%)	64
Total	468 (53.6%)	378 (43.0%)	33 (3.4%)	879

Patients who were treated with alternative femoral implants and cemented femoral fixation, owing to markedly reduced bone quality, and patients with all types of revision arthroplasty were excluded.

**Fig. 2:**
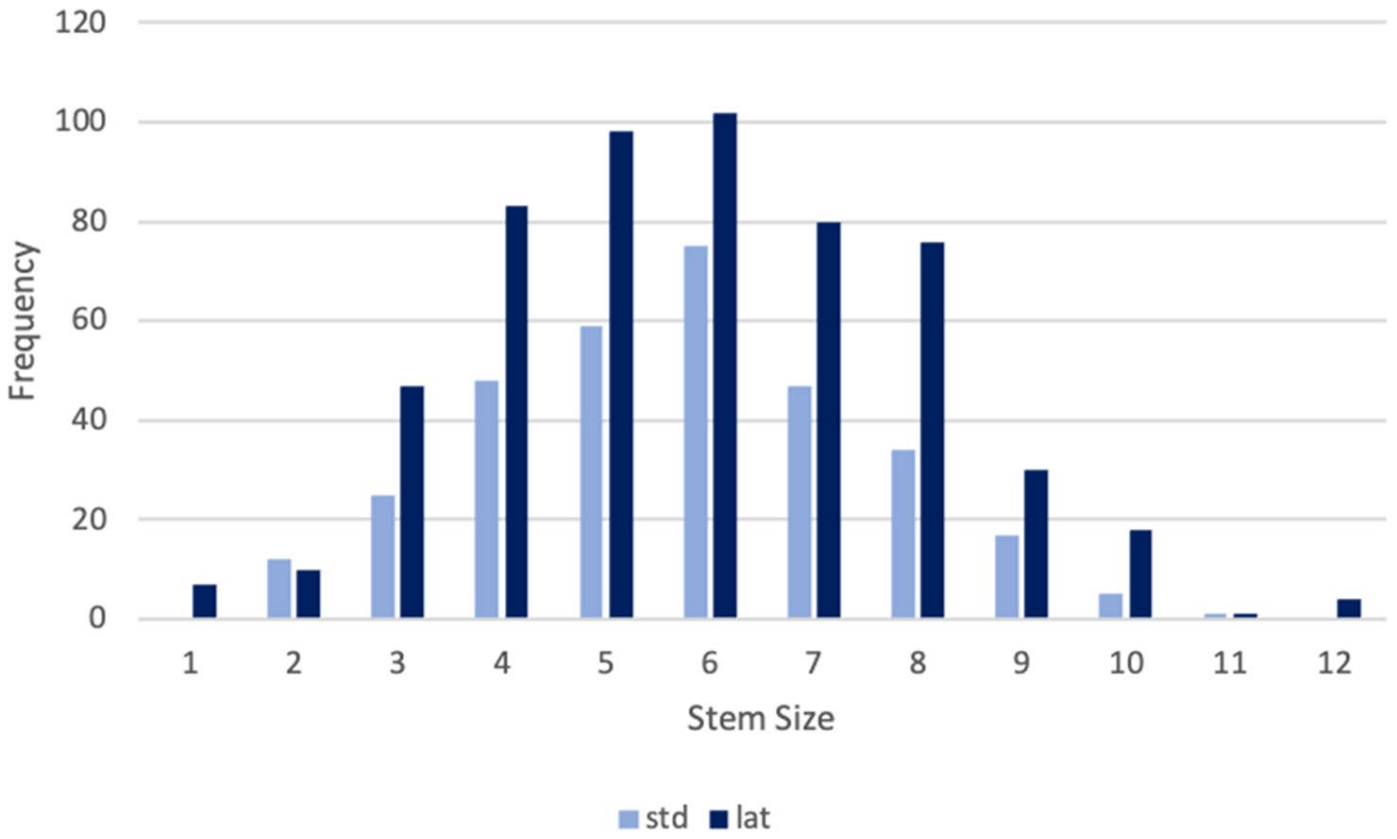
Frequency of different stem sizes and offset versions used

In all patients, the meta-diaphyseal anchoring, calcar-guided optimys stem (Mathys Ltd. Bettlach, Switzerland) was used (Fig. 1). It is a monobloc short stem with an osteoinductive titanium plasma spray and calcium phosphate coating, for which a standard and lateralized neck is available. It has been classified type 2B in the classification system of Khanuja et al. [1]. Fig 2 shows the frequency of different stem sizes implanted (Fig. 2). In 323 hips, a standard neck and in 556 hips a lateralized neck was used.

The optimys stem was combined with various cementless press-fit cups using either a ceramic–polyethylene or ceramic–ceramic bearing couple, according to the surgeon’s preferences. In a few cases, a cemented polyethylene cup was used. Almost all patients received a pure alumina (Al_2_O_3_) ceramic head type Bionit2 (Mathys Ltd., Bettlach, Switzerland). In 247 hips, the femoral head diameter used was 28 mm, whereas a 32 mm diameter was used in 133 hips and a 36 mm was used in 499 hips.

A total of three different approaches were applied. The majority of implantations were performed using the antero-lateral approach in the supine position (80.2%); the next two most common approaches were the direct anterior approach (17.9%) and the transgluteal approach in the lateral position (1.9%) (Table 2).

**Table 2:** Distribution of different approaches

Clinic	Approach			
	Anterior	Antero-lateral	Transgluteal (lateral)	Total
Center 1	0 0.0%	216 100.0%	0 0.0%	216
Center 2	8 2.9%	256 91.7%	15 5.4%	279
Center 3	0 0.0%	233 99.1%	2 0.9%	235
Center 4	85 100.0%	0 0.0%	0 0.0%	85
Center 5	64 100.0%	0 0.0%	0 0.0%	64
Total	157 17.9%	705 80.2%	17 1.9%	879

Postoperative care was predetermined according to each center’s guidelines. However, all patients were allowed to engage in fully weight bearing activities after surgery, except when an intraoperative complication had occurred.

After surgery, a standardized follow-up protocol was conducted with timepoints at 6 weeks, 6 months, 12 months, 24 months, and 5 years.

Rates and reasons for complications and revisions were documented during follow-up and analyzed at the last follow-up.

At every timepoint, PROMs were obtained, including the Harris Hip Score (HHS), as well as pain and satisfaction, which were measured using a visual analog scale.

### Statistical methods

Descriptive statistics included mean, median, standard deviation, and ranges. Between-group differences were evaluated using nonparametric tests: the 2-samples Wilcoxon test and the Kruskal–Wallis test, the latter for cases of more than two groups. Associations between discrete variables were carried out using chi-square tests. In the case of sparse data, Fisher’s exact test was used. Tests of differences from the baseline were evaluated using paired *t* tests. Survival analyses of stem survival regarding different strata, such as center, sex, age class, body mass index (BMI) class, and surgical approach were conducted using the Kaplan–Meier method, censoring patients at death or last follow-up date. The 95% confidence intervals for survival rates were calculated using the log–log method. Log-rank tests were used for group comparisons. In addition, Cox proportional hazard regression models were used to assess survival risks of these strata variables simultaneously. The level of significance was set at *p* = 0.05 (two-sided) for all tests.

All statistical analyses were performed using SAS version 9.4 (SAS Institute Inc., Cary, NC, USA).

## Results

Across 5 participating centers, 782 eligible patients (49.8% women and 50.2% men) who underwent a total of 879 THAs were included. The mean age was 64.8 years (SD 10.4). Patients had a mean BMI of 28.1 (SD 5.1). Across centers, patients did not differ by sex or BMI; however, there were significant differences in mean patient age (*p* < 0.001) (Table 3). The distribution of Dorr types is shown in Table 1. Again, significant differences were observed across centers (*p* < 0.0001) (Table 1).

**Table 3:** Patient demographics

Statistics	Age	Height	Weight	BMI
Center 1				
*n*	216	216	216	216
Mean (SD)	62.9 (9.80)	172.9 (8.22)	83.5 (16.99)	27.9 (5.22)
95% CI	61.6, 64.2	171.8, 174.0	81.2, 85.7	27.2, 28.6
Median	63.5	172.0	82.0	26.9
Range	33–88	152–192	50–153	19–45
Center 2				
*n*	279	279	279	279
Mean (SD)	64.8 (11.36)	171.4 (9.13)	83.4 (16.60)	28.4 (5.16)
95% CI	63.5, 66.1	170.3, 172.4	81.5, 85.4	27.8, 29.0
Median	66.5	171.5	82.0	27.5
Range	24–91	148–195	47–164	18–53
Center 3				
*n*	235	235	235	235
Mean (SD)	66.5 (9.97)	169.8 (8.96)	82.1 (16.15)	28.4 (4.83)
95% CI	65.2, 67.8	168.7, 171.0	80.0, 84.1	27.8, 29.0
Median	68.3	170.0	82.0	28.0
Range	34–87	150–192	44–165	16–49
Center 4				
*n*	85	85	85	85
Mean (SD)	63.8 (10.08)	171.1 (8.15)	82.9 (17.21)	28.3 (5.49)
95% CI	61.6, 66.0	169.3, 172.8	79.2, 86.6	27.1, 29.5
Median	65.4	172.0	80.0	26.3
Range	38–88	152–193	50–150	19–47
Center 5				
*n*	64	64	64	64
Mean (SD)	67.0 (9.27)	168.5 (8.20)	76.0 (14.47)	26.7 (4.49)
95% CI	64.7, 69.3	166.4, 170.5	72.3, 79.6	25.6, 27.8
Median	66.2	168.5	75.0	26.1
Range	43–88	152–186	42–103	16–37
Total				
*n*	879	879	879	879
Mean (SD)	64.8 (10.44)	171.1 (8.79)	82.5 (16.57)	28.1 (5.08)
95% CI	64.1, 65.5	170.5, 171.7	81.4, 83.6	27.8, 28.5
Median	66.3	170.0	81.0	27.4
Range	24–91	148–195	42–165	16–53
Kruskal–Wallis Test (DF = 4)	*p* = 0.00078	*p* = 0.0024	*p* = 0.070	*p* = 0.072

Whereas small stem sizes ≤ 6 were mostly used in Dorr type A femora, in Dorr type B and C the majority of stems used were size > 6. Those associations proved to be highly significant (*p = *0.00018).

The majority of patients had a preoperative diagnosis of primary or secondary osteoarthritis. Ninety-seven patients underwent bilateral hip arthroplasty.

A total of 42 patients died in the study cohort of 782 patients. All of these deaths were not related to the index surgery.

**Fig. 3:**
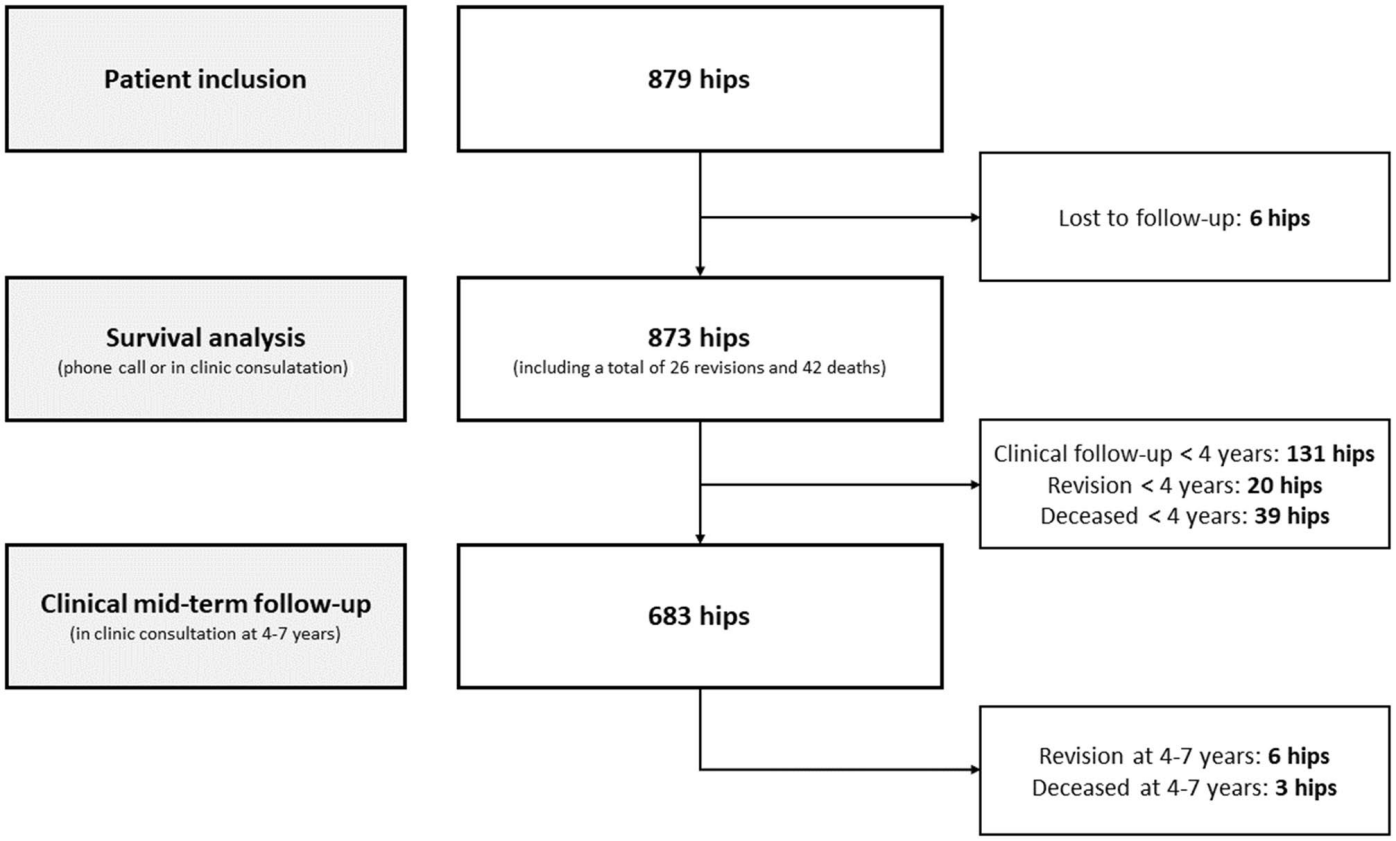
Patient flow

The patient flow is shown in Fig. 3. For 131 hips, an in-clinic consultation at 4–7 years could not be accomplished, but information was obtained by phone call. These hips are not included in the clinical follow-up but are included in the survival analysis. At a mean of 64.3 months (SD 6.1), 683/879 THAs (77.7%) were clinically assessed by in-clinic consultation in person with a minimum of 48 months of follow-up. At 4–7 years of follow-up, another six hips needed revision surgery and another three patients with three hips died (Fig. 3).

### Complications

Table 4 details the local complications excluding periprosthetic fractures. A high rate of intraoperative acetabular perforations was observed, owing to issues with one particular cup design in Center 3. Apart from this, the most common local complications were hematoma, seroma, and femoral nerve palsy.

**Table 4:** Local complications (%)

	Intraoperative	During hospital stay	During follow-up
Acetabulum perforation	2.7	0.3	0.1
Incorrect implantation of cup	0.1		
Haematoma/seroma		3.1	0.3
Femoral nerve palsy		0.8	0.2
Superficial infection		0.1	0.5
Wound healing disorder		0.1	0.3
Dislocation		0.1	0.2
Vasular lesion		0.1	
Wound dehiscence		0.1	
Thigh pain			0.3

A total of 20 intraoperative and postoperative periprosthetic femoral fractures were observed, including proximal fissures and avulsion fractures of the greater trochanter, of which 11 were successfully treated conservatively (Table 5). Periprosthetic fractures were observed in 0.9% of Dorr type A femora, in 3.5% of Dorr type B femora, and in 9.7% of Dorr type C femora (*p* = 0.00084).

**Table 5:** Periprosthetic femoral fractures and fissures

	Femoral fissure	Avulsion of greater trochanter	Periprosthetic femoral fracture
Intraoperative			
No treatment	3	3	
Cerclage/Wiring	3		1
During follow-up			
Conservative treatment	1		4
Stem revision			5

All periprosthetic fractures occurred in patients where the anterior or antero-lateral approach was used. Due to the small numbers, no statistical significance was found (*p* = 0.878). Furthermore, no significant influence of the different stem sizes on the occurrence of periprosthetic fractures was found (*p* = 0.583).

### Revisions

**Fig. 4:**
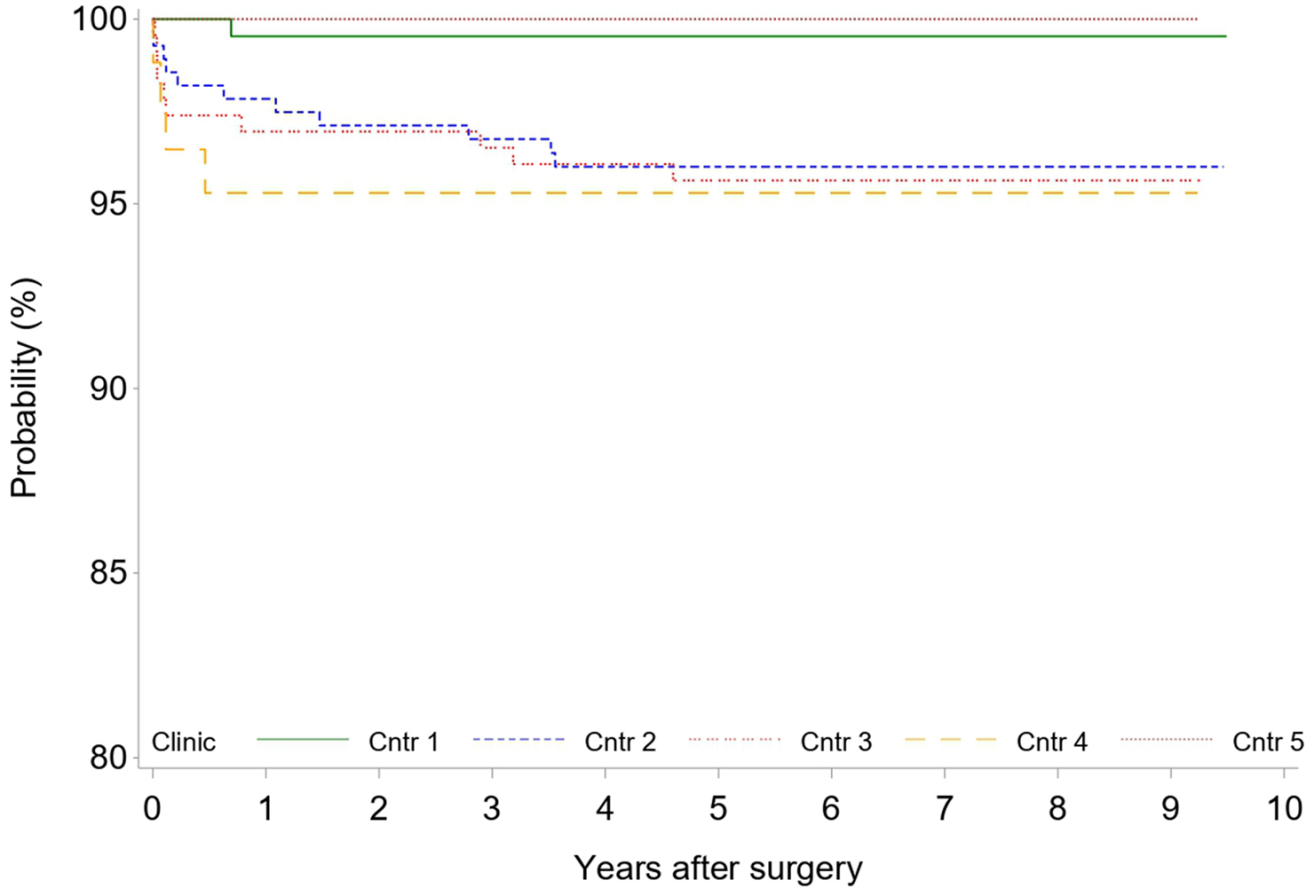
Kaplan–Meier survival plot for any revision by clinic

A total of 26 patients (3.0%) required at least one revision after the index procedure. To date, a total of 28 revisions were performed. Regression analysis suggested a significant association between the need for revision surgery and center (center effect) (*p* = 0.0414) (Fig. 4). As previously reported in the short-term results [7], in six hips, the head and inlay were changed owing to early infection. However, one of those cases presented during mid-term follow-up as late-onset infection at 55 months, requiring two-stage exchange of all components, including the stem. Besides infection, the main reasons for revision surgery without the necessity of revising the stem were malpositioning of the acetabular component with periprosthetic acetabular fractures (five cases) and fractures of the ceramic head (two cases).

**Fig. 5:**
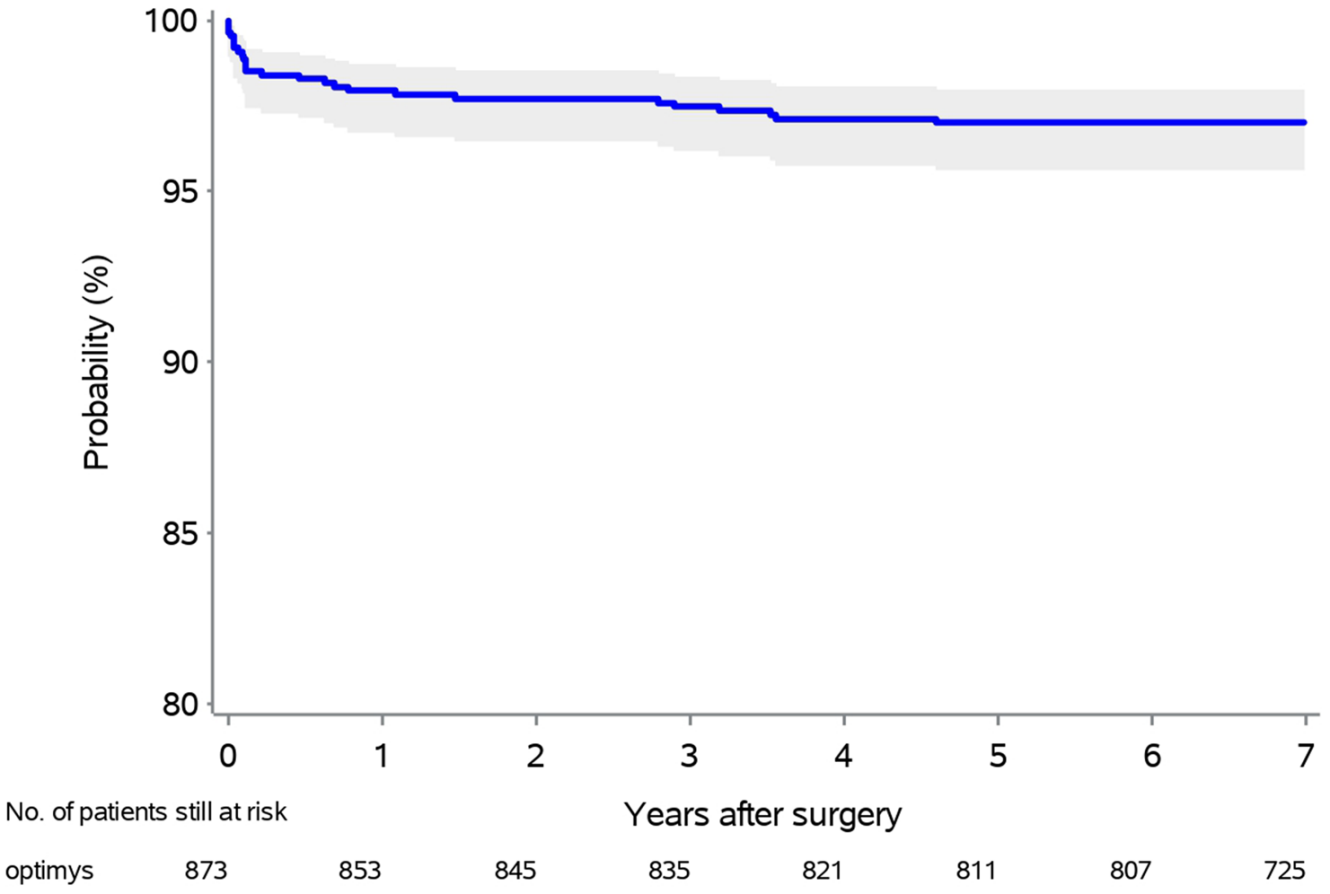
Kaplan–Meier survival plot for stem revision

**Fig. 6:**
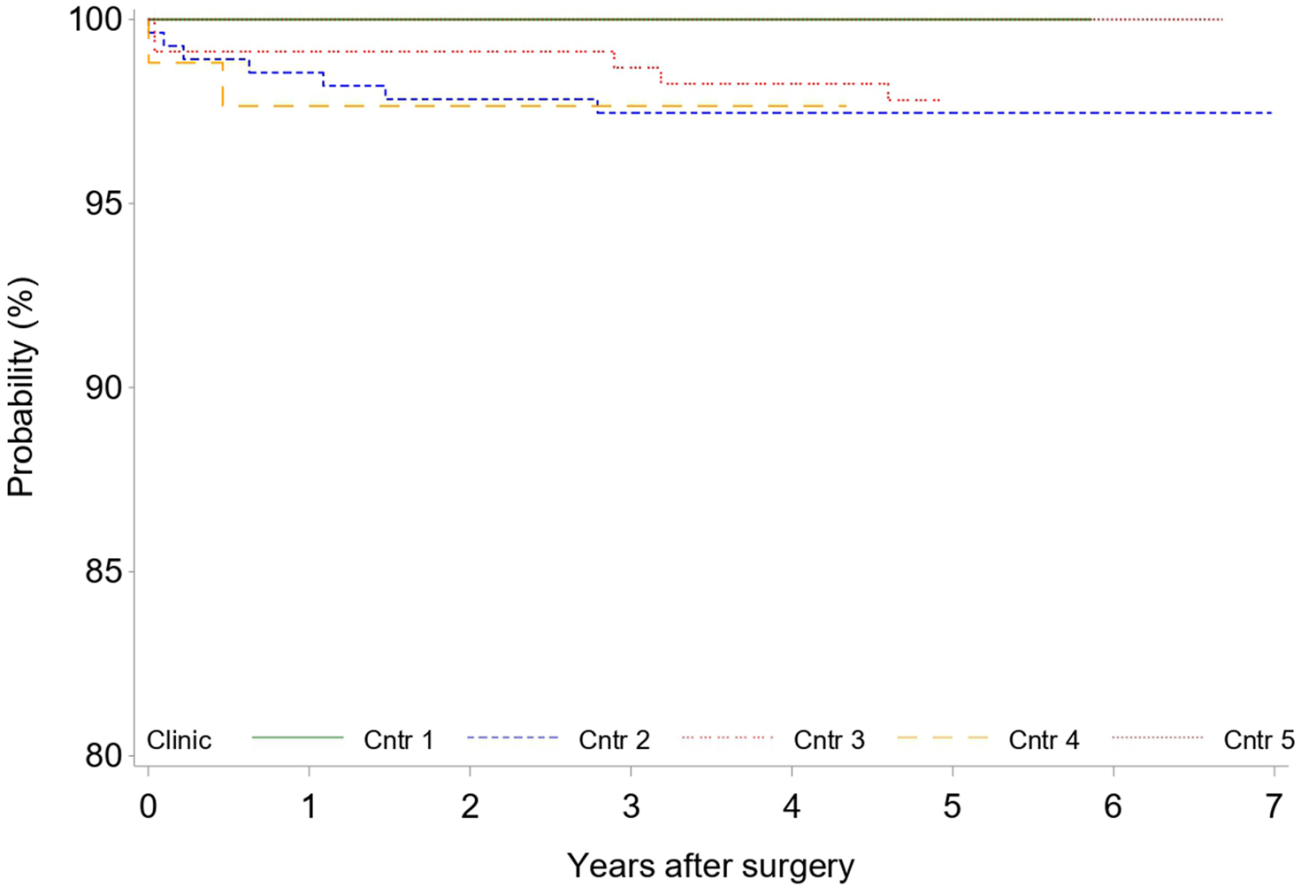
Kaplan–Meier survival plot of stem revision by clinic

A total of 14 stem revisions (1.6%) had to be performed at mid-term. Overall, the survival rate for endpoint stem revision at 6 years was 98.4% (Fig. 5). Five stems presented with aseptic loosening, there were five cases of early periprosthetic femoral fractures, three infections required revision of all components, and in one case the stem was reinserted after revision of the cup. Regression analysis revealed no significant association between the need for stem revision and center (*p* = 0.1458) (Fig. 6).

**Fig. 7:**
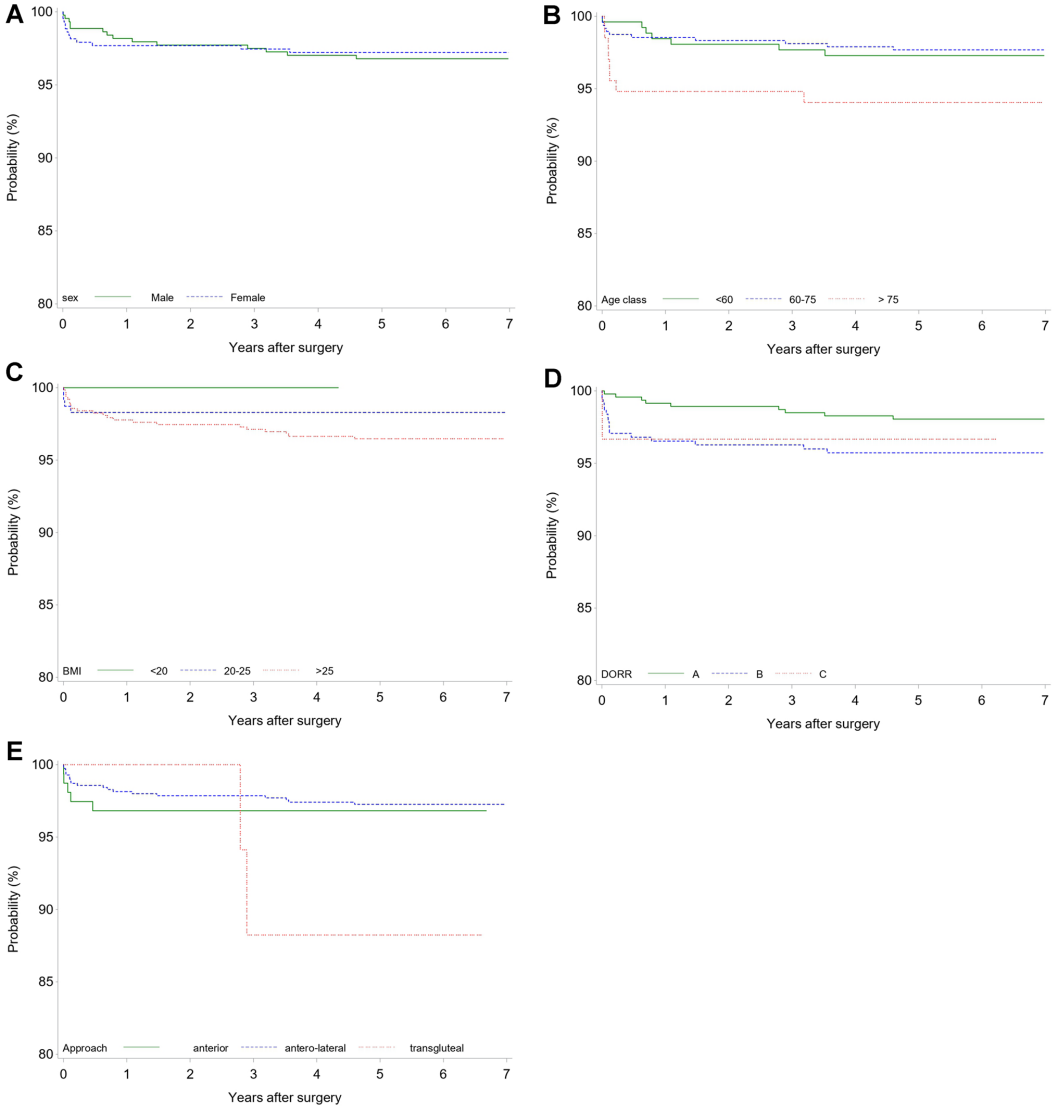
Kaplan–Meier survival plot of stem revision by: **A** sex; **B** age class; **C** BMI class; **D** Dorr classification; **E** approach

Kaplan–Meier survival plots analyzing the influence of sex, age, BMI, Dorr type, and different approaches on stem survival are given in Fig. 7A–E. Whereas sex had no influence on stem survival, older patients and patients with a high BMI showed an increased risk for stem revision during follow-up; however, this was not statistically significant (*p* = 0.0806 and *p* = 0.3372). Dorr type A versus revealed significantly lower risk of stem revision than Dorr type B or C (*p* = 0.0465). The transgluteal approach was associated with a lower stem survival rate compared with the anterior and the antero-lateral approach although given the small numbers of operations using the transgluteal approach, a statistically robust conclusion is not possible (*p* = 0.1002).

### Patient-reported outcome measures

All PROMs are summarized in Table 6. Across centers, at last follow-up, significant differences were found regarding HHS (*p* < 0.0001), satisfaction (*p* = 0.023), and load pain (*p* < 0.0001).

**Table 6:** Patient-reported outcome measurements at baseline and follow-up

Statistics	Baseline				At last follow-up			
	HHS	Satisfaction	Rest pain	Load pain	HHS	Satisfaction	Rest pain	Load pain
Center 1								
Mean (SD)	45.6 (15.84)	1.9 (2.18)	5.2 (3.00)	7.7 (2.18)	97.8 (4.70)	9.7 (0.71)	0.1 (0.46)	0.6 (1.16)
Median	47.0	1.0	5.0	8.0	100.0	10.0	0.0	0.0
Range	7–88	0–10	0–10	0–10	65–100	6–10	0–3	0–5
Center 2								
Mean (SD)	47.3 (12.87)	1.1 (1.85)	4.5 (2.82)	7.3 (2.12)	95.2 (10.62)	9.5 (1.23)	0.2 (0.89)	0.8 (1.56)
Median	48.0	0.0	5.0	8.0	99.0	10.0	0.0	0.0
Range	8–81	0–10	0–10	0–10	23–100	2–10	0–8	0–9
Center 3								
Mean (SD)	41.7 (13.67)	4.6 (1.54)	4.3 (2.23)	7.9 (1.15)	95.1 (8.69)	9.8 (0.66)	0.1 (0.44)	0.2 (0.63)
Median	44.0	5.0	4.0	8.0	99.0	10.0	0.0	0.0
Range	8–70	0–8	0–10	4–10	49–100	4–10	0–5	0–6
Center 4								
Mean (SD)	61.7 (13.83)	3.6 (1.63)	5.5 (2.15)	6.6 (1.58)	97.5 (6.82)	9.7 (0.85)	0.1 (0.44)	0.3 (1.12)
Median	62.0	3.0	6.0	7.0	100.0	10.0	0.0	0.0
Range	42 to 92	1–9	0–9	1–9	62–100	5–10	0–3	0–7
Center 5								
Mean (SD)	56.1 (9.35)	4.0 (1.24)	2.8 (1.93)	6.2 (1.10)	99.3 (1.30)	10.0 (0.30)	0.0 (0.00)	0.0 (0.21)
Median	58.0	4.0	2.0	6.0	100.0	10.0	0.0	0.0
Range	38–76	2–6	0–8	4–8	96–100	8–10	0–0	0–1
Total								
Mean (SD)	47.5 (15.10)	2.6 (2.32)	4.6 (2.70)	7.4 (1.90)	96.5 (8.04)	9.7 (0.89)	0.1 (0.60)	0.5 (1.18)
Median	48.0	2.0	5.0	8.0	100.0	10.0	0.0	0.0
Range	7–92	0–10	0–10	0–10	23–100	2–10	0–8	0–9
Kruskal–Wallis Test	*p* < 0.0001	*p* < 0.0001	*p* < 0.0001	*p* < 0.0001	*p* < 0.0001	*p* = 0.023	*p* = 0.110	*p* < 0.0001

## Discussion

The outcomes of the present prospective multicenter observational study demonstrate that calcar-guided short-stem THA using the optimys stem results in satisfactory survivorship at mid-term follow-up, given an all-cause stem revision rate of 1.6% (100–98.4%) at 6 years. Clinical results were excellent.

Given that many conventional stems in THA have already proven excellent long-term results in THA, for new implants to be accepted, they must be equally safe and effective as existing implant designs. At the same time, they should potentially offer some benefits to the surgeon, such as increased ease of implantation or easier revision [10]. Short-stem THA has been reported to enhance the intraoperative safety. For example, Molli et al. found a lower rate of intraoperative complications, including fractures, in a series of 606 patients who underwent THA with a short stem compared with those receiving a conventional stem (0.4% vs. 3.1%, respectively) [11]. Hochreiter et al. compared blood loss and transfusion rate of short-stem THA using the optimys stem against conventional THA. Both blood loss and blood transfusion rates were found to be lower in patients after short stem THA, compared with conventional stem THA [12]. Given the short and rounded design, short stems have the additional advantage of easier insertion when using minimally invasive approaches for THA, again, potentially sparing soft tissue and further reducing blood loss [13, 14]. However, the implantation technique differs from conventional THA and most probably involves a severe learning curve [5, 15].

To date, it remains unknown whether this new group of femoral implants will perform as well as conventional stems in terms of revision rate and implant survival in the long term. In a recent review of national registry data, together with case studies, a revision rate of approximately 5% after 10 years was found for several conservative implants [16]. Another systematic review and meta-analysis of randomized controlled trials, comparing short-stem THA with conventional THA, suggested that short-stem THA may provide superior bone remodeling and, at the same time, deliver similar survival rates and clinical outcomes [17]. However, in both reviews, the short-term follow-up of the included studies was inadequate for determining the long-term performance of short stems. Already in 2014, van Oldenrijk et al. systematically reviewed the revision rates of short-stem THA including 49 studies, demonstrating favorable medium-term revision rates [18]. In conclusion, the need to conduct long-term controlled studies as well was emphasized, since the majority of the included studies had a follow-up of less than 5 years. In those meta-analyses, a large number of different stem designs was involved. Clinical studies of so-called “collum stems” or “neck retaining stems” did not demonstrate satisfactory survival rates, while low revision rates were found for all other short-stem designs. Only two studies regarding the optimys stem, as a type of so-called “partial collum” or “femoral neck sparing” stem, were part of those reviews. A small collective study with a follow-up of only 6 months was included [19]. One single revision resulted in a revision rate per 100 observed component years of 3.2 [18]. This stem revision was, however, required as the result of a periprosthetic fracture after a fall by an older patient with dementia. A second collective, of 204 cases with a mean follow-up of 30.4 months, resulted in one revision due to infection; however, it was not necessary to revise the stem [20]. ***blinded.

Only in recent years, have short stems been involved in national arthroplasty registries. When looking at the results of several national registries, contemporary short stems are among the most successful implants, regarding implant survival at early stages. For example, in the German national joint registry (Das Endoprothesenregister Deutschland), new-generation calcar-guided short stems, such as the optimys stem, the NANOS stem (Smith & Nephew, Marl, Germany), the A2 stem (Artiqo, Luedinghausen, Germany), and the MiniHip stem (Corin, Cirencester, UK) have been associated with excellent implant survival. A revision rate of 2.1% at 5 years has been documented for the optimys stem [21]. These data are strongly supported by the Australian and the Swiss national registries, which give similar results for these implant design [22, 23]. However, again, only registry data of a maximum of 5 years of follow-up are available. Therefore, it is of the utmost importance to gather further data on the long-term survival of contemporary short-stem designs.

The stem revision rate observed in this multicenter investigation was 1.6%, resulting in a Kaplan–Meier survival of 98.4% at 6 years, thus, confirming the registry data.

Besides some cases of deep periprosthetic infection, requiring revision of all components, most stem revisions were due to aseptic loosening and periprosthetic fractures. It appears that there is a quite substantial variation in survival rates across the centers. However, as some centers have only a few revisions, a statistical evaluation is far from robust. A potential difference in stem revision rate might be explained by the fact that some of the participating centers were less experienced in using earlier short-stem designs before the initiation of the study than others. It is further worth noting that revisions occurred rather early after surgery for those centers having a rather high overall revision rate. Given that the implantations of the present investigation had been the first ones ever using the optimys stem worldwide, a learning curve was involved. There might have been surgical mistakes intraoperatively, leading to early failure in some cases. Additionally, patient demographics of the included cases were significantly different across the five centers. Indications for short-stem THA may have been decided on differently. For example, the mean age ranged from 62.9 years in center 1 to 67.0 years in center 5. At the same time, the mean BMI of the included patients ranged from 26.7 to 28.4 kg/m^2^. It is well known that revision rates in conventional THA are highly influenced by different age and BMI group [24, 25]. Also, previously published data of the present collective suggested an increased rate of periprosthetic fractures in older patients [7]. This can be confirmed by the present analyses since, particularly in the group of patients aged > 75 years, early periprosthetic fractures led to an inferior Kaplan–Meier survival compared with younger groups, resulting in the main drop of the curve during the first months. Again, the main risk factor for the incidence of early periprosthetic fractures was found to be reduced bone quality. Whereas in Dorr type A and B femora a low fracture risk was observed, giving a fracture rate of 9.7%, the usage of calcar-guided short stems in Dorr type C femora cannot be recommended [8]. Although all periprosthetic fractures occurred using the anterior or the antero-lateral approach, no statistical association was observed. At the same time, in the younger groups, stem revisions particularly had to be performed as a result of aseptic loosening, which is demonstrated through a steady but mild drop of the survival curve over the entire follow-up period. Intraoperative undersizing of the stem, resulting in continuous migration over the first years postoperatively, may be considered one of the main reasons for this observation [26–28]. Regarding periprosthetic fractures no correlation with different stem sizes were obvious. Patients with high BMI showed the most inferior implant survival; however, no statistically significant differences were found, compared with patients with low or medium BMI. Interestingly, in 2 cases out of only 17 implantations using the transgluteal lateral approach in the whole collective, a stem revision was needed, leading to a stem survival of only 87.5% after 3 years. Given the small numbers of the transgluteal approach, however, no valid conclusion can be made regarding this finding.

The strength of this study lies in the prospective multicenter design with a large number of included hips. Furthermore, given that it comprises the first implantations of this particular stem worldwide, the present data include the learning curve. Even better outcomes regarding complications and early revisions can be expected for surgeons with broad experience with the investigated stem design. However, some limitations have to be acknowledged. First, the distributions of indications and patient’s demographics across the centers were not identical. It is obvious that patient-related factors, such as age or bone quality may have had impact on the surgeon’s choice of implant. The collective, however, represents a useful cross-section of patients. Additionally, this allowed for a statistical comparison of the outcomes of different centers and may help to confirm adequate indications and contraindications for this stem design. Second, whereas for the survival analysis almost all included hips were considered, a clinical examination in person could be only performed of 683 hips. However, at 4–7 years, for 873 hips, follow-up information could be obtained, either in person at the clinic or through a phone call. Finally, to date, only mid-term data could be obtained. Although the mid-term results are highly promising, it is not clear whether these results will also be sustained in the long term.

In conclusion, the optimys short stem produced highly satisfactory clinical outcomes at mid-term, with 98.4% implant survival for any cause of stem revision and low rates of complications.

Insertion of this stem requires an exacting technique to prevent the potential pitfalls of incorrect stem sizing and intraoperative fracture.

Long-term results are required to further evaluate the stem’s promising mid-term results.

## References

[1] Khanuja HS, Banerjee S, Jain D, Pivec R, Mont MA (2014). Short bone-conserving stems in cementless hip arthroplasty. J Bone Joint Surg Am.

[2] Hauer G, Vielgut I, Amerstorfer F, Maurer-Ertl W, Leithner A, Sadoghi P (2018). Survival rate of short-stem hip prostheses: a comparative analysis of clinical studies and national arthroplasty registers. J Arthroplasty.

[3] Kutzner KP, Pfeil J (2018). Individualized stem-positioning in calcar-guided short-stem total hip arthroplasty. J Vis Exp.

[4] Kutzner KP, Freitag T, Donner S, Kovacevic MP, Bieger R (2017). Outcome of extensive varus and valgus stem alignment in short-stem THA: clinical and radiological analysis using EBRA-FCA. Arch Orthop Trauma Surg.

[5] Kutzner KP, Donner S, Loweg L, Rehbein P, Dargel J, Drees P (2019). Mid-term results of a new-generation calcar-guided short stem in THA: clinical and radiological 5-year follow-up of 216 cases. J Orthop Traumatol.

[6] Mai S, Pfeil J, Siebert W, Kutzner KP (2016). Kalkar-geführte Kurzschäfte in der Hüftendoprothetik - eine Übersicht. OUP.

[7] Camenzind RS, Kutzner KP, Mai S, Juch F, Bosson D, Helmy N (2020) Clinical and radiological short-term results for a calcar guided short-stem: multicentre study of 879 cases. Acta Orthop Belg; 86(eSuppl(3)):40–48.

[8] Gkagkalis G, Goetti P, Mai S, Meinecke I, Helmy N, Bosson D, Kutzner KP (2019) Cementless short-stem total hip arthroplasty in the elderly patient - is it a safe option? A prospective multicentre observational study. BMC Geriatr; Apr 17;19(1):112. 10.1186/s12877-019-1123-1. 10.1186/s12877-019-1123-1 PMC6472082 30995903

[9] Dorr LD, Faugere MC, Mackel AM, Gruen TA, Bognar B, Malluche HH (1993). Structural and cellular assessment of bone quality of proximal femur. Bone.

[10] Blakeney WG, Lavigne M, Beaulieu Y, Puliero B, Vendittoli PA (2021). Mid-term results of total hip arthroplasty using a novel uncemented short femoral stem with metaphyso-diaphyseal fixation. Hip Int.

[11] Molli RG, Lombardi AV, Berend KR, Adams JB, Sneller MA (2012). A short tapered stem reduces intraoperative complications in primary total hip arthroplasty. Clin Orthop Relat Res.

[12] Hochreiter J, Hejkrlik W, Emmanuel K, Hitzl W, Ortmaier R (2016). Blood loss and transfusion rate in short stem hip arthroplasty. A comparative study. Int Orthop.

[13] Lombardi AV, Berend KR, Ng VY (2011). Stubby stems: good things come in small packages. Orthopedics.

[14] Kutzner KP, Hechtner M, Pfeil D, Rehbein P, Kovacevic MP, Schneider M (2017). Incidence of heterotopic ossification in minimally invasive short-stem THA using the modified anterolateral approach. Hip Int.

[15] Loweg L, Kutzner KP, Trost M, Hechtner M, Drees P, Pfeil J (2017). The learning curve in short-stem THA: influence of the surgeon’s experience on intraoperative adjustments due to intraoperative radiography. Eur J Orthop Surg Traumatol.

[16] Evans JT, Evans JP, Walker RW, Blom AW, Whitehouse MR, Sayers A (2019). How long does a hip replacement last? A systematic review and meta-analysis of case series and national registry reports with more than 15 years of follow-up. Lancet.

[17] Liang H-D, Yang W-Y, Pan J-K, Huang H-T, Luo M-H, Zeng L-F (2018). Are short-stem prostheses superior to conventional stem prostheses in primary total hip arthroplasty? A systematic review and meta-analysis of randomised controlled trials. BMJ Open.

[18] van Oldenrijk J, Molleman J, Klaver M, Poolman RW, Haverkamp D (2014). Revision rate after short-stem total hip arthroplasty: a systematic review of 49 studies. Acta Orthop.

[19] Pfeil J, Grieshaber SW, H, , Jerosch J (2012). Optimys. Kurzschaftendoprothesen - Wo liegen die Unterschiede?.

[20] Kutzner KP, Pfeil D, Kovacevic MP, Rehbein P, Mai S, Siebert W (2016). Radiographic alterations in short-stem total hip arthroplasty: a 2-year follow-up study of 216 cases. Hip Int.

[21] German Arthroplasty Registry (EPRD): Annual Report 2020. 2020. https://www.eprd.de. Accessed 01 Oct 2021

[22] Australian Orthopaedic Association National Joint Replacement Registry. 2020. https://www.aoanjrr.sahmri.com. Accessed 01 Oct 2021

[23] Annual Report of the Swiss National Joint Registry (SIRIS), Hip and Knee, 2012–2019. 2020. https://www.swiss-medtech.ch. Accessed 01 Oct 2021

[24] Bayliss LE, Culliford D, Monk AP, Glyn-Jones S, Prieto-Alhambra D, Judge A (2017). The effect of patient age at intervention on risk of implant revision after total replacement of the hip or knee: a population-based cohort study. Lancet.

[25] Sayed-Noor AS, Mukka S, Mohaddes M, Kärrholm J, Rolfson O (2019). Body mass index is associated with risk of reoperation and revision after primary total hip arthroplasty: a study of the Swedish Hip Arthroplasty Register including 83,146 patients. Acta Orthop.

[26] Schaer MO, Finsterwald M, Holweg I, Dimitriou D, Antoniadis A, Helmy N (2019). Migration analysis of a metaphyseal-anchored short femoral stem in cementless THA and factors affecting the stem subsidence. BMC Musculoskelet Disord.

[27] Kutzner KP, Ried E, Donner S, Bieger R, Pfeil J, Freitag T (2020). Mid-term migration pattern of a calcar-guided short stem: a five-year EBRA-FCA-study. J Orthop Sci.

[28] Kutzner KP, Freitag T, Bieger R (2020). Defining “undersizing” in short-stem total hip arthroplasty: the importance of sufficient contact with the lateral femoral cortex. Hip Int.

